# Superior Performance of Magnetic Nanoparticles for Entrapment and Fixation of Bovine Serum Albumin In-Vitro

**DOI:** 10.4314/ejhs.v30i4.15

**Published:** 2020-07-01

**Authors:** Mansour Binandeh, Farrokh Karimi, Sadegh Rostamnia

**Affiliations:** 1 Faculty of Science, Department of Chemistry, University of Maragheh, Iran; 2 Faculty of Science, Department of Biotechnology and Enviromental, University of Maragheh, Iran

**Keywords:** Magnetic nanoparticles, Spectrophotometer and Electrophoresis analysis, Electrostatic bonding

## Abstract

**Background:**

In recent years, extensive studies have been performed on magnetite nanoparticles (MNPs) and their applications, which have shown the current project to be one of the major applications by laboratory results.

**Methods:**

The nanoparticles synthesized in this project were deposited by the co-precipitation method, which structure was identified by analyzers such as SEM, FT-IR, and EDX. The aim of this project is the adsorption and fixation of biomolecule (BSA (bovine serum albumin) protein on the surface of magnetic nanoparticles.

**Results:**

The adsorption results by electrophoresis and spectrophotometric analyzers showed an absorption rate above 55% ie; 55% of the protein is fixed on the MNPs nanoparticles. This absorption is due to the high level of functionality of magnetic nanoparticles for adsorption of protein. The results of the EDX analysis also show the possible electrostatic bonding between the nanoparticles and the protein, this is derived from –OH with –NH_2_ groups of the nanobiocompound (MNPs /protein). After bonding, the two are easily separated.

**Conclusion:**

In this project, the Fe_3_O_4_ nanoparticles was synthesized and identified by SEM, FT-IR, and EDX analyzers and finally reacted with the BSA protein (for the absorption of protein on MNPs) under experimental conditions at a standard temperature of 25° C. The results showed that about 55% of the protein was fixed on magnetic nanoparticles.

## Introduction

Nanotechnology is a key element in understanding the nature of the coming decade. Interdisciplinary research collaboration, special training and transfer of ideas from people in the industry, including the benefits of nanotechnology is required in the future. Nanocatalysts nanotechnology industry had been made significant progress and, among them, iron oxide nanoparticles (based on magnetic properties) had been many applications especially in the field of drug delivery, the gene and proteins are necessary. One of the most important and most widely used magnetic nanoparticles in which a variety of materials to create their unique characteristics compared to other Nano-specific applications screwed. These particles are applied in various branches. But the role of them in life-medicine and, as mentioned, is significant in terms of its delivery to the inherent magnetism gives them a lot of things, including facilitating spotter the delivery of these are very important (1).

### Application and structure of magnetic nanoparticles:

Over the past few years, efforts have been devoted to the magnetic Functionalized nanoparticles as the level of cover will gain significant benefits from it. However, there are many types of materials available in magnetic coatings Nanoparticles, such as metal oxides, metal, and plastic is still considered to be the best candidate surfaces Functionalization because it is highly stable against degradation. Also, nanoparticles have biocompatibility that is used for various applications in recent years, such as separation of protein and enzyme immobilization (2–3, 15–19).

### The method of stabilizing BSA protein on the surface of magnetite magnetic nanoparticles:

One of the main techniques is that biomolecular techniques have now been established on magnetic nanoparticles. These biochemical molecules and biological biochemicals (4), proteins (5) are important for the treatment and stabilization by magnetic nanoparticles. In this project, biomolecules (protein) are discussed. One of the main issues is the creation of the relationship between biomolecules (6,10) and the catalyst stabilized with magnetic nanoparticles. Usually, an electrostatic bond between magnetic nanoparticles and biomolecules, and in some cases, can also be a covalent bond depending on the magnetic nanoparticle ligands (7).

### Outlook of magnetic nanoparticle application in-vitro:

**Figure 1:**
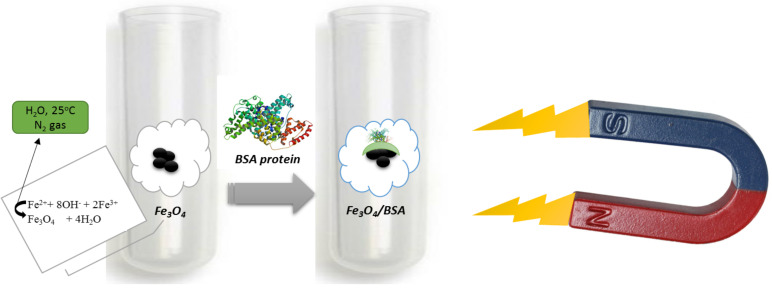
General route for the synthesis of Fe_3_O_4_ magnetic nanoparticles which reacted with the protein

Proteins conjugated with large microparticles made of gelatin or polyglutaraldehyde improves it levels when interacting with agar electrophoretic detection. This is called a targeted medicinal and its absorption efficacy has improved. To improve this type of adsorption, magnetic nanoparticles smaller than 20 nm must first be synthesized in order to achieve the exact method proposed by this project (ie, the mechanism of magnetic nanoparticle synthesis) in such a way that the size of the nanoparticles is precise with core/shell structure measured to be useful for medicinal absorption and stabilization purposes. Therefore, magnetic nanoparticles smaller than 20 nm are suitable for a variety of biosorbent reactions with a variety of biomolecules as well as for bactericidal (8–9), which was synthesized in this project to be specifically used for BSA protein. There are many papers and researchers to stabilize biomolecules on magnetic nanoparticles. The main goal of this project is Fe3O4 magnetic nanoparticles for the stabilization of biomolecules (protein (BSA) (serum albumin)) biomolecules. In this project, we intend to introduce a synthetic mechanism by obtaining an appropriate fixation of the protein on the surface of the nanoparticles (at room temperature and fully sterilized), to investigate by EDX analysis (Figure 1).

## Methods

All solvents and chemicals are purchased from commercial suppliers. The structure of materials was provided by the transmission electron microscope (Philips CM-200 and Titan Krios TEM, (Mohageg Ardabil University). BSA (bovine serum albumin) were obtained from Sigma (St. Louis, MO). Materials such as; ferrous (ii) chloride tetrahydrate, ferric (iii) nitride Nona hydrate and sodium hydroxide were purchased from Merck KGaA (Darmstadt, Germany). And, phosphate-buffered saline (PBS (pHs 6.0–8.0)), argon gas, HCl, methanol, TritonX100, EDTA, Boric acid, NaCl, glutaraldehyde and Salmon sperm (protein) sodium salt is purchased from Sino-pharm Chemical Reagent Co. (Shanghai, China). The protein (BSA) used in the lab is models (Maragheh University, Iran). Deionized water was used in each experiment.

### Synthesis of Fe_3_O_4_ magnetic nanoparticles:

Different mechanisms have been designed for the synthesis of hollow magnetite microspheres (8,12). Chemical Co-precipitation also one of the easiest and most convenient methods of synthesis of magnetic nanoparticles with core/shell structure. The method is determined by the following formula M the same amount of iron salts (II). M=Fe^2+^, Fe^3+^=2Fe^2+^

**Figure:**
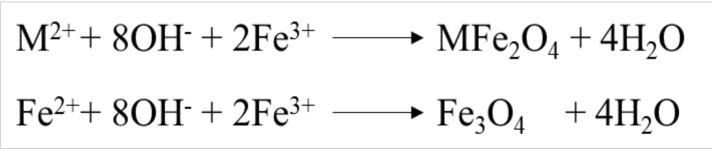


Thus, in this way, sample container iron salts with amounts of 1 to 2 (90mmoles of FeCl_2_.4H_2_O and 180mmoles of Fe (NO_3_)_3_.9H_2_O) were dissolved in distilled water. The reaction temperature was 25° C and high-intensity spinning under inert nitrogen gas. Finally, the yellowish-brown product was obtained in the same magnetic nanoparticles. Finally, the solution was washed repeatedly with methanol and water and then dried in the oven since the powder was gathered (Fe_3_O_4_ magnetic nanoparticles).

### BSA adsorption from aqueous solution:

BSA adsorption experiments were carried out in batch-wise. Approximately 25mg of magnetic nanoparticles were mixed with 1ml of various concentrations (but optimum concentration was made) of BSA solution in water. The mixture was shaken at room temperature (25° C) for 0.5 h, which proved to be a sufficient period to reach equilibrium. Then the magnetic nanoparticles were separated with help of the permanent magnet and the supernatant was assayed for remaining protein concentration by the UV–Vis spectrophotometer at 595 nm.

### Regeneration studies:

BSA desorption experiments were performed in a binding buffer containing various concentrations of PBS. BSA adsorbed particles were placed in the desorption medium and stirred at 25° C for 0.5 h. The ratio desorption and absorption were calculated for the BSA.

## Results

### Synthesis and characterization of magnetic nanoparticle:

**Figure 2:**
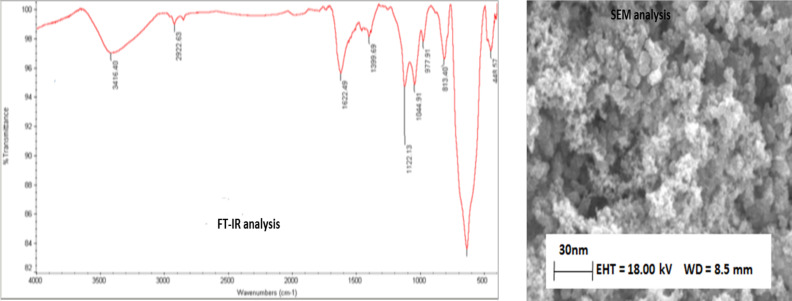
SEM and FT-IR analyzes for structure detection of Fe_3_O_4_ magnetic nanoparticles

For the preparation of magnetic nanoparticles of magnetite (Fe_3_O_4_) to iron metal oxide from the oxide, Fe^+2^, Fe^+3^ were chemical co-precipitation way. In the range of 10–50nm magnetic nanoparticles should be made, such as the size of the magnetic nanoparticles in chemical reactions and medical procedures are important. To investigate and establish the structure of the magnetic nanoparticles are used in a series of analyzes, including; TEM, EDX, SEM, XRD, FT-IR, TGA and so on. The project analyzed by SEM, FT-IR, and EDX was used to stabilize the structure of magnetite magnetic nanoparticles. Analytical SEM, analysis of a series of images of the structure of the magnetic core/shell nanoparticles provided. Analysis FTIR, the analysis to show the operating groups, and also established a covalent bond between functional groups. It values in terms of frequency from 500 to 4000 cm^−1^ are based on. The FT-IR spectra of as-prepared hollow magnetite microspheres were characterized by a high absorption band at 802 cm^−1^ imputed to the typical band of Fe_3_O_4_, equivalent to the stretching vibration modes of Fe-O (11,12). Results for identifying the structure of magnetic nanoparticles by functional groups shown in (Figure 2).

### Adsorption studies:

As the project title suggests, I have tried that to stable biomolecules such as a protein on magnetic nanoparticles Fe_3_O_4_. So, I just have to expand other researchers in this area, that I have achieved a basic conclusion. As you know, the magnetic nanoparticles and their magnetic properties are able to be controlled remotely by an external magnetic field. Before must, biomolecules of interest (protein) in response to MNPs that, to be a link between them. The most important thing is the phenomenon of absorption of (protein) on MNPs. It was bonding, an electrostatic bond for (protein) which was taken of the EDX analysis (13).

### Results of protein loaded onto magnetic nanoparticles Fe_3_O_4_ by spectrophotometry:

The purpose of this section is to investigate the absorption of drug biomolecules on the magnetic nanoparticle bed. Therefore, using the equation (8–9), we can examine the absorption rate. Under standard conditions, the amount of 20 micrograms per µl of BSA protein is dissolved in 1 ml of sterile water, and then 25 milligrams of magnetic nanoparticles weigh in one separate dishe and on BSA protein (at time optimal, 30min) was solved.

**Figure 3:**
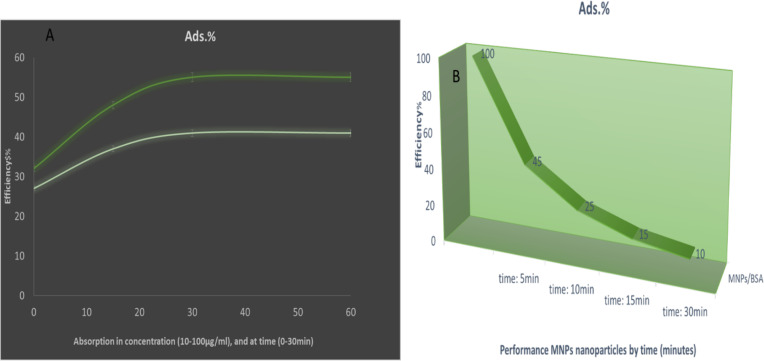
A) Results of protein adsorption on magnetic nanoparticles at concentrations of (0–100µg/ml), and time at (0–60 minutes), B) Results of protein removing of on the surface of the MNPs by PBS solution

The instant of dissolution of the mixture was continued at the instant of zero minutes to about an hour, that summarized the obtained data. The results showed that the absorption rate of the protein in the first half hour was about 55%. The results obtained in the future did not change later, and so this the absorption between MNPs and BSA is one bonding, It has been proved by the analysis described in the intended section (based on the results obtained from EDX analysis). The results of the absorption rate (analyzed at concentrations of 10–100 µg/mL, which is the optimum concentration of 20 µg/mL) are shown in the (Figure 3A). The time required to perform this process is described below.

### Efficiency of time:

Efficiency time for absorption, 0.5h in this test as the standard time for this research is very important. At the beginning of the reaction, patterns were to study the magnetic nanoparticles@protein absorption studies conducted showed that the uptake protein the concentration (20 µg/ml), in The wavelength of 595nm (absorption wavelength protein) is 55% and after 0.5h absorption be stable over time because the absorption process of protein was complete to stable on the surface of magnetic nanoparticles. The absorption of BSA protein within a half-hour episode was tested and the results were observed in (Figure 3A). And according to the results, we find that over time gradually increased uptake, and it been stable after 0.5h for protein.

### Stabilization and release process of protein on MNPs:

The absorption evaluation protein on magnetic nanoparticles, the amount of 1µg. ml^−1^ (protein) is standard was developed by Bradford formula (OD=2, C=1µg. ml^−1^). Which is defined as follows; 1) **C=50.OD/100**

In formulas 50 and 100, there are fixed numbers and OD (absorbance). OD (absorbance value as well as Beer-Lambert law (14)) can be calculated and measured by spectrophotometer analysis. The law of beer Lambert states that part of the light after coloring with glass solution, absorption, and other passes. Continue to work with the device by a spectrophotometer, 25mg amount of magnetic nanoparticles were added to a 1 mL (BSA protein) solution. Finally, the absorbance it on the surface of the magnetic nanoparticles was measured by samples in optical tubes separated and analyzed within 0 to 30 min of time. For this action, 100µl of MNPs/BSA protein nanocomposite was prepared in 400 µl samples at a time interval of 0–30 minutes was analyzed. All samples were isolated from the solution at the desired time collected and prepared for analysis. Adsorption rate at different concentrations (10–100 µg/ml) was analyzed by the spectrophotometer UV-VIs. All samples were taken and ready for use in a device with a wavelength (595 nm for protein) which absorption rate was measured, the PBS buffer solution (buffer containing 20mM phosphate buffer in pHs 6.0–8.0, it was dissolved in 1M NaCl) was used to obtain the amount of protein fixed on the nanoparticles, which washed all the protein from the surface of the magnetic nanoparticles and analyzed only the remaining protein from the original compound within the cell (at intervals of 0–30 min). According to data, the absorption of protein on magnetic nanoparticles is 55%. The results showed that more than 90% of the total (protein) was removed from the surface of the MNPs nanoparticles by PBS solution, so all the quantities shown in (Table 1, Figure 3B). The results of the discussion are that absorption 20µg/ml is maximum and so, it was above this value of 100µg/ml, the absorption gradient has not been altered. The data are shown in the following (Figure 3A) and the measured values of the standard scale are considered.

**Table 1:**
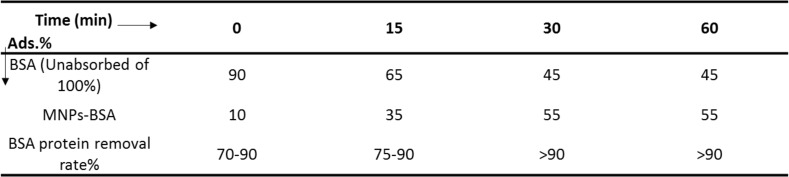
Contains data showing the efficiency of all the magnetic nanoparticles' efficiency

### Results of electrophoresis analysis:

**Figure 4:**
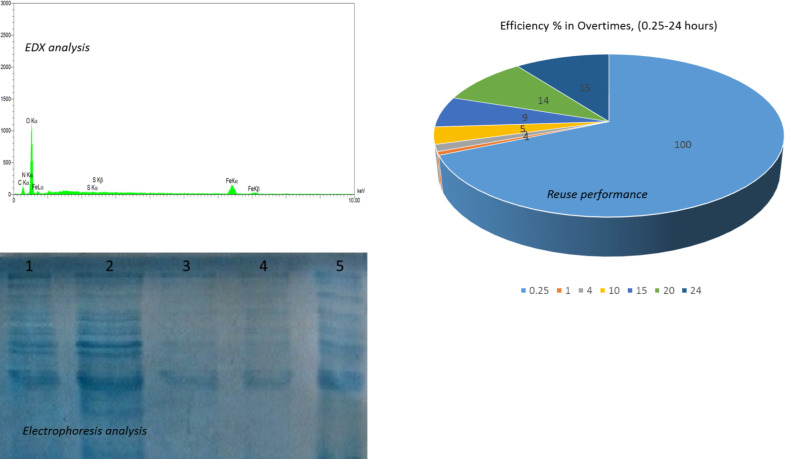
Electrophoresis and EDX analyzes were performed to stabilize the amount of protein adsorbed on magnetic nanoparticles, and reuse efficiency was used to evaluate the nanoparticles' performance over a period of 0.25–24 h

In this section, protein is analyzed by electrophoresis. Electrophoresis analysis is based on absorption at absorption timing. Here, vertical electrophoresis to measure protein absorption. With respect to (Figure 4), it can be seen that the number of stained specimens in the range of 0 to 30 min of times, the absorption of protein on the magnetic nanoparticles have gradually dimmed, which this fading stain shows that biomolecules are absorbed on the surface of magnetic nanoparticles. Therefore, spectrophotometric and electrophoresis of both devices showed acceptable results for this absorption. First, in 5 different patterns for protein in three separate tests at a dose of 20µg/ml and at 0–30min on a sol-gel plate (Different mixed supernatant solution and MNPs/BSA protein). In the first, first point Electrophoresis analysis was ladder used to marking for identifying the amount of protein adsorbed on the magnetic nanoparticle substrate. First, we put the previously prepared agar jelly plate on a clean glass so that it is perfectly smooth and without slope. Then we stained it at certain intervals. In total, we put 5 spots that are as follows: in the first line is the ladder and the second line is spot of pure protein, the third of nanoparticles and protein at zero minutes (the moment when no reaction between the two), and the fourth (15min) and fifth (30min) spots afterward. The electrophoresis machine then began to scan, isolate, and detect the amount of protein present in the compounds at different times. After 2–5 hours, the results showed that the brightness and size of the three to fifth spots were lower than the second spot (pure BSA protein, unabsorbed). Based on these results, it can be demonstrated that the protein stabilized on the nanoparticles for up to 30 minutes and after this time, there was no change in absorbance (55%).

We studied a mixture of protein-MNPs, which biomolecules (protein) were dissolved together with magnetic nanoparticles in distilled water. The results of the adsorptions were analyzed in the electrophoresis, and the results showed that the concentration of biomolecule in the solution decreased with time of 0–30 minutes. Using spectrophotometric analysis, it was proved that this reduction process means absorption to the surface of magnetic nanoparticles. Finally, it can be proven that a bond between the biomolecule and the surface of the nanoparticles is carried out, which is one bonding that was measured by the EDX analysis, in section next.

### Results of EDX analysis:

In this discussion, by W% of elements perceived whichever were dependent on the reactants of protein and magnetic nanoparticles Fe_3_O_4_. EDX analysis showed that both of the reactants bonded together been in the product. Also, elements of Fek_α_ and Fek_β_ with O (with a strong peak) is shown. This analysis may be a demonstrative bond between magnetic nanoparticles Fe_3_O_4_ and BSA. The results of the EDX analysis show that binding of agent N (-NH_2_ group of protein) in 750 keV and agent of O (-OH group of MNPs) in 1100 keV because they are in a line and so, it may be stated this approaching is electrostatic bonding same for N-O. Element O is in the agent group H_2_O element N of the agent NH_2_ of protein. Evidence of EDX analysis is a Spectrophotometer seconder for this tissue. The result of absorption, and the link between magnetic nanoparticles and BSA protein by EDX analysis, shown in (Figure 4).

### Stability of magnetic nanoparticles in repeated use after recycling:

The results reusing of the magnetic nanoparticles for BSA adsorptions were analyzed by spectrophotometric analysis over a period of 1–24 h for 7 periods. Results showed that the efficiency performance of nanoparticles in the application again for the stabilization of biomolecules, that is only about a 10–15% reduction in power, indicating the efficiency and high ability of magnetic nanoparticles to stabilize multiple of BSA protein. The magnitude of this stability has been investigated at different times (the reaction process and optimal conditions are the same as in the discussion and conclusion). The results are shown in (Figure 4).

## Discussion

In this section, very important issues are discussed. One is that the magnetic nanoparticles (MNPs) are synthesized by combining of combining two iron salts (II, III), in a ratio of 1 to 2 in distilled water by chemical co-precipitation method, and are prepared in the form of a yellowish-brown powder dried in oven. Some of this powder is analysed by SEM analyses (to check the exact structure of regular crystals and particle size in the range of 20–100 nm), FT-IR (study of functional groups to form an interconnected structure based on the wave number, which is 802 cm^−1^) (8, 9).

25 mg of it (MNPs) was weighed and dissolved in distilled water, then 20 micro L/ml of BSA was added to it and the reaction was performed at room temperature (25°C) on a heat-free heater. Over a period of 0–30 minutes, several samples were removed (By placing a magnet under the reaction vessel, the MNPs/BSA were absorbed into the field and 20 microliters were removed from the top solution). These samples were measured by a spectrophotometric device, and the results showed that the absorption of BSA on the surface of MNPs was 55% (8, 9).

Another way to prove this absorption is to study the bond created between MNPs and BSA nanoparticles by EDX device (which is based on the amount of elements of compound and numbers close together indicate on bond between the atoms, ie at 750 keV) (13). Therefore, the existence of an electrostatic bond between Fe-O elements from MNPs with NH from BSA was established (8, 9). This system is made (by a magnetic field) for targeted transmission (15–19).

In conclusion, this project is one of the special applications of magnetic nanoparticles in the medical industry. Although this work has been done in vitro, but by using recombinant technique in genetics, the protein stabilized on the surface of the magnetic nanoparticles can be targeted to an external magnetic field control (in-vivo with injection 25micrograms/dL into the rat vein) equipped, then the protein delivered to the target receptor in the damaged cell (under the control of sympathetic and parasympathetic nerves via a neurotransmitter such as epinephrine or acetylcholine) to synaptically localize the brain and the spinal cord attaches to the target receptor (the process will continue in the in-vivo test) and finally, the nanoparticle by external magnetic fields of magnetic and under the guidance of the protein targeted cells outside the body to be transferred. So this is a completely scientific and curable method that can ultimately be done in an ashley large, specialized way for the treatment of damaged cells in humans.
